# 27.2 THE DOPAMINE MOTIVE SYSTEM IN ADDICTION

**DOI:** 10.1093/schbul/sby014.112

**Published:** 2018-04-01

**Authors:** Nora Volkow

**Affiliations:** DHHS/National Institute on Drug Abuse

## Abstract

**Background:**

We have investigated the role of bidirectional interactions between the dopamine reward and motivation system and executive function in addicted individuals, with a particular focus on the intersection between the role of D2 receptor (D2R) signaling in the striatum and perturbations in prefrontal brain activity.

**Methods:**

Using brain imaging we have studied these interaction for various types of addiction and explored how their involvement affect behavior including impulsivity and compulsiveness. We have also investigated the mechanisms associated with vulnerability to drug use disorders as linked with disrupted executive function including the effects of genetics and physiological factors such as circadian rhythms, sleep deprivation and obesity.

**Results:**

We found that: a) chronic drug use reduces striatal levels of D2R and perturbs metabolism in frontal brain regions, emotional reactivity and executive control; b) that higher-than-normal striatal D2R availability in nonalcoholic members of alcoholic families appear to play a protective role against alcoholism by regulating circuits involved in inhibiting behavioral responses and in controlling emotions; c) that chronic sleep deprivation is associated with increased striatal dopamine, lower D2R availability, and metabolic changes in several cortical brain regions; and, d) that newly characterized variable number tandem repeat (VNTR) polymorphisms in the genes coding for PER2 and the AKT1 proteins (a kinase that has been implicated in schizophrenia and psychosis) appear to modulate striatal D2R availability in the human brain.

**Discussion:**

Although the studies have focused on the effects of drugs, the DA striato cortical pathway is of direct relevance to schizophrenia as well as that of other psychiatric disorders. We will discuss the implications of our findings as they relate to the prevention and treatment of substance use disorders and schizophrenia.

